# Combinatorial targeting of menin and the histone methyltransferase DOT1L as a novel therapeutic strategy for treatment of chemotherapy-resistant ovarian cancer

**DOI:** 10.1186/s12935-022-02740-6

**Published:** 2022-11-04

**Authors:** Elena Alexandrova, Jessica Lamberti, Domenico Memoli, Claudia Quercia, Viola Melone, Francesca Rizzo, Roberta Tarallo, Giorgio Giurato, Giovanni Nassa, Alessandro Weisz

**Affiliations:** 1 Laboratory of Molecular Medicine and Genomics, Department of Medicine, Surgery and Dentistry ’Scuola Medica Salernitana’, University of Salerno, 84081 Baronissi, Italy; 2 Medical Genomics Program, AOU ‘S. Giovanni di Dio e Ruggi d’Aragona’ University of Salerno and Rete Oncologica Campana, 84131 Salerno, Italy; 3 Genome Research Center for Health - CRGS, Campus of Medicine of the University of Salerno, 84081 Baronissi, Italy

**Keywords:** Ovarian cancer, Menin, DOT1L, Drug combination, Chemotherapy resistance

## Abstract

**Background:**

Ovarian cancer (OC) is characterized by a low response rate and high frequency of resistance development to currently available treatments. The therapeutic potential of histone methyltransferase DOT1L inhibitor in OC cells has been demonstrated, but optimal efficacy and safety of this targeted therapy approach still require improvement. We set forth to evaluate if this problem can be overcome by combinatorial targeting of this epigenetic modifier and menin, one of its functional partners in chromatin.

**Methods:**

siRNA-mediated gene knock-down and pharmacological inhibition of menin, a key component of the MLL/SET1 complex and a fitness gene in OC cells, coupled to cell proliferation assays on a panel of high grade serous OC cell lines, including chemotherapy-sensitive and -resistant clones, were applied in order to evaluate how depletion or blockade of this enzyme influences growth and viability of OC cells. RNA sequencing was applied to identify menin target genes and pathways, and the effects of combined inhibition of menin and DOT1L on growth and transcriptome of these OC models were evaluated.

**Results:**

Silencing and pharmacological inhibition of menin exert antiproliferative effects in all OC cells tested and, in PEO1 and PEO4 cells, a profound impact on transcriptome *via* down-regulation of cell cycle regulatory pathways, aryl hydrocarbon receptor, MYC and KRAS signalling. We demonstrated association of menin and DOT1L in OC cells and identified a subset of genes co-regulated by the two factors. Interestingly, co-treatment with DOT1L and menin pharmacological inhibitors exerts an additive effect on growth inhibition on chemotherapy-sensitive and -refractory OC cells mediated by transcriptome changes controlled by menin and DOT1L activities.

**Conclusion:**

These results indicate that menin functionally cooperates with DOT1L in OC cells modulating transcription of genes involved in key cellular functions including, among others, cell proliferation and survival, that are strongly affected by combined inhibition of these two epigenetic regulators, suggesting that this may represent a novel therapeutic strategy for chemotherapy-resistant OCs.

**Trial registration:**

NA; The manuscript does not contain clinical trials.

**Supplementary Information:**

The online version contains supplementary material available at 10.1186/s12935-022-02740-6.

## Introduction

Ovarian cancer (OC) is the leading cause of death from gynaecological malignancies, accounting for 313,959 new cases in 2020 and more than 200,000 victims [1]. Due to the lack of specific symptoms, OC is frequently diagnosed at advanced stages and is characterized by low response rate and the high frequency of resistance to current treatments [2]. These tumours are highly heterogeneous and classified into several subtypes, each characterized by distinct gene expression, epigenetic and mutational patterns, and, consequently, critically differing one from another. Among OCs, high-grade serous ovarian cancer (HGSOC) represents the most common, aggressive and lethal form of epithelial ovarian cancer [3], pointing out to an urgent need to identify novel therapeutic targets and treatment approaches for this tumour subtype.

Our group recently demonstrated that the histone methyltransferase DOT1L represents an effective therapeutic target in ERα-expressing OC cells where pharmacological blockade of this epigenetic enzyme induces inhibition of estrogen signalling [4]. Other studies highlighted ERα-independent DOT1L role in OC mediated by transcriptional regulation of cell cycle [5] and multi-drug resistance genes [6] and suggested DOT1L as a valuable prognostic biomarker in OC. Evaluation of therapeutic potential of DOT1L pharmacological inhibition has proved to be effective in treatment of multiple cancer types including ovarian, breast, prostate and other solid tumours (reviewed in [7]). However, DOT1L inhibitors use in clinics is still hampered by low tolerance and severe side effects caused by drug administration indicating to the need of therapeutic efficacy improvement for these epigenetic drugs [7]. Therefore, new therapies are being sought to allow the doses of DOT1L-targeting drugs to be reduced, including application of drug combination treatments.

Most of the proteins in a cell do not act alone but through physical interaction with multiple co-factors, form multiprotein complexes that govern cellular processes. Thus, targeting components of protein assemblies containing factors known to exert mitogenic activity in cancer cells may represent a plausible strategy for novel drug target discovery for treatment of this malignancy [8]. Moreover, simultaneous inhibition of activity of proteins belonging to the same molecular complex have potential to enhance targeted therapy effectiveness and safety using the sub-optimal drug concentrations [9].

Scaffold protein menin, encoded by multiple endocrine neoplasia 1 gene (*MEN1*) and known to be involved in histone modification and epigenetic gene regulation, has been shown to physically bind DOT1L in leukaemia and breast cancer cells [10], [11]. Moreover, menin interaction with MLL is involved in development of acute leukaemias with translocations of the *MLL* gene [12], [13], as well as of solid tumours, including castration-resistant prostate cancer [14] and hepatocellular carcinoma [15]. Importantly, menin-MLL inhibitors are currently being clinically tested for relapsed or refractory acute myeloid leukaemias treatment and therefore may soon be introduced in clinical practice for treatment of this disease, reducing time required for its approval for treatment of other cancer types.

In the present study we characterized the effects of menin silencing and its pharmacological inhibition on the proliferation of a panel of HGSOC cells and extensively investigated transcriptional changes induced by its depletion and blockade in chemotherapy-sensitive and resistant relapsed OC models. We showed here that menin targeting may be effective in reinforcement of DOT1L inhibition effect since their cooperative blockade in OC cells has additive antiproliferative effect.

## Materials and methods

### Ovarian tissue and cell lines data analysis

Data for 264 ovarian cancers and 180 normal ovarian tissue samples were collected from web resources ICGC [16] and GTEx, respectively, and were used for comparative analysis of *MEN1* mRNA expression level in cancer and normal tissues (derived from normal regions of the left and or/ right ovary of women that got traumatic injury, cerebrovascular, heart, liver, renal, respiratory or neurological diseases). CRISPR-Cas9 gene knock-down data were analysed using DepMap portal (https://score.depmap.sanger.ac.uk).

### Cell lines

Ovarian cancer cell lines PEO1 (ECACC 10032308), PEO4 (ECACC 10032309), PEO14 (ECACC 10032311) were purchased from the American European Collection of Authenticated Cell Cultures (ECACC, Salisbury, UK), whereas ovarian cancer cell lines OVCAR-3 (HTB-161) and Caov-3 (HTB-75) were ordered from American Type Culture Collection (ATCC, Manassas, VA, USA). Cells were cultivated according to manufacturers’ instructions at 37 °C and in the presence of 5% CO_2_. ATCC formulated RPMI 1640 medium (Euroclone, Milan, Italy), supplemented with 10% FBS (GE Healthcare, Chicago, IL) was used for maintenance of PEO1, PEO4, and PEO14 cell lines. RPMI 1640 medium supplemented with 20% FBS and 0.01 mg/ml bovine insulin (Merck, Darmstadt, Germany) was used for cultivation of OVCAR-3 cells. Caov-3 cells were grown in DMEM (Euroclone) supplemented with 10% FBS. For all cell lines, 100 U/ml penicillin (Lonza, Basel, Switzerland), 100 mg/mL streptomycin (Lonza), and 250 ng/mL Amphotericin-B (Merck) were added to the culture medium. Mycoplasma contamination was routinely tested using a mycoplasma PCR detection kit (ABM, Richmond, BC, Canada).

### Transient small interfering RNA transfection

Reverse transfection of *MEN1*-specific siRNAs (IDs: s8682, s8683, and s8684), targeting different gene regions, and scrambled negative control (ID: s813), all purchased from Ambion (Thermo Fisher Scientific, Austin, TX, USA), was performed into all cell lines using Lipofectamine RNAiMAX (Invitrogen, Thermo Fisher Scientific), according to the manufacturer’s instructions. First, 0,25 µl of 2 pM siRNA were diluted in 4,75 µl of Opti-MEM reduced serum medium (Gibco, Thermo Fisher Scientific), supplemented with 100 U/ml penicillin, 100 mg/mL streptomycin, and 250 ng/mL Amphotericin-B. Then, a mix of 0.3 µl of Lipofectamine RNAiMAX and 4,7 µl of Opti-MEM medium was added to diluted siRNA (1:1 v/v ratio), vortexed and incubated for 30 min at room temperature. At the end of incubation, 40 µl of Opti-MEM medium was added to the mix, and 50 µl of the final master mix was aliquoted in a well of 96-well TPP tissue culture plate (Merck). Finally, 15*10^3^ cells resuspended in a 50 µl of Opti-MEM medium were plated on the top of siRNA-lipid complex. Cells were incubated for 4 h at 37 °C in the presence of 5% CO_2_. After that, 100 µl of RPMI (in case of PEO1, PEO4, PEO14 and OVCAR-3 cells) or DMEM (in case of Caov-3 cells), medium supplemented with 20 or 40% FBS were added to each well reaching a final volume of 200 µl. Each treatment was performed in sextuplicate. After 96 h, cells were harvested for protein, RNA extraction or used for measurement of cell viability by MTT assay.

### Compounds

All cell lines were incubated with increasing concentrations of the menin-MLL interaction inhibitors MI-136 (S7815) or MI-503 (S7817), both purchased from Selleckchem (Houston, TX, USA) or vehicle (DMSO, Merck) as control. PEO1 and PEO4 cell lines were exposed to a combination of MI-136 and DOT1L inhibitor EPZ5676 (S7062, Selleckchem), at the indicated concentrations or vehicle (DMSO) as control.

### Cell proliferation analysis

96 h post-transfection, cell proliferation was evaluated by MTT (3-(4,5-Dimethylthiazol-2-yr)-2,5-Diphenyltetrazolium Bromide)-based colorimetric assay (Invitrogen, Carlsbad, CA), according to the manufacturer instructions. Absorbance was measured by the VICTOR Multilabel Plate Reader (PerkinElmer, Milan, Italy) at 570 nm wavelength, subtracting background values read at 620 nm wavelengths for each sample. In order to assess the effects of the compounds on cell growth, 3*10^3^ cells per well were seeded in sextuplicate in a 96-well TPP tissue culture plate. Cell proliferation was determined as described above after cells exposure to drugs, drugs combination or vehicle for 3, 6, 9, and 12 days.

### Total protein extraction

For total protein extraction, cells were harvested, washed twice with ice-cold PBS-EDTA (0.5 mM EDTA), and lysed for 15 min on ice in high salt buffer (Tris-HCl pH 7.5 50 mM, NaCl 180 mM, NP40 0.15%, Glycerol 10%, MgCl2 1.5 mM, NaMo4 1mM, and NaF 0.5 mM). At the end of incubation, samples were centrifuged at 13.000 rpm for 30 min at 4 °C, and the supernatant containing total protein extract was diluted with two volumes of low salt buffer (Tris-HCl pH 7.5 50 mM, NP40 0.15%, Glycerol 10%). Protein concentration was measured using a Bradford protein assay.

### Immunoprecipitation

For immunoprecipitation, 1 mg PEO4 nuclear protein extract prepared as it was previously described [17], was incubated overnight at 4 °C with 35 µl of anti-rabbit IgG-coated Magnetic Beads (Thermo Fisher Scientific), pre-conjugated for 4 h at 4 °C with 2 µg of anti-menin antibody (A300-105A, Thermo Fisher Scientific) or Rabbit IgG Isotype Control (02-6102, Thermo Fisher Scientific) antibody as a negative control. At the end of the incubation, beads were washed as described in [18], resuspended in sample buffer (0.167 M Tris-HCl pH 6.8, 10% glycerol, 4% SDS, 3,1% DTT and 0.004% Bromphenol blue) and boiled at 100 °C for 5 min for protein elution.

### Western blotting

SDS-PAGE and Western blotting analyses were performed using standard protocols. For protein detection, the following primary antibodies according to their manufacturer’s instructions were used: mouse monoclonal anti-β-actin (A1978, Sigma-Aldrich, St Louis, MO); rabbit polyclonal anti-menin (A300-105A, Thermo Fisher Scientific) and anti-KMT4/Dot1L (ab72454, Abcam, Cambridge, UK).

### Total RNA extraction

Total RNA was extracted from all cell lines using TRIzol reagent (Life Technologies, Carlsbad, CA), according to the manufacturer’s instructions. In case of siRNA-transfected cells, total RNA samples both for RT-qPCR and RNA sequencing were extracted 96 h post transfection using 50 µl of TRIzol reagent per well of 96-well plate. All treatments were performed in sextuplicate, pooled together and further processed as unique sample following TRIzol extraction protocol until phase separation step after which the supernatant was collected and further purified using RNA Clean & Concentrator − 5 kit (Zymo Research, Irvine, CA, USA) according to the manufacturer’s instructions. Two independent biological replicates were prepared for both *MEN1*-targeting siRNA and scramble control. RNA extraction from PEO1 and PEO4 cells treated with 3.2 µM EPZ5676, 0.8 µM MI-136, their combination, or vehicle was performed after nine-day cultivation of OC cells in the presence of the indicated compounds. Three independent biological replicates were prepared for all treatments and for validation experiments. Before use, RNA purity was assessed by NanoDrop™ 2000/2000c spectrophotometer (Thermo Fisher Scientific), whereas its concentration and integrity were measured using Qubit RNA assay kit and fluorimeter (Life Technologies) and Agilent 4200 Tapestation System (Agilent, Santa Clara, CA, USA), respectively.

### RT-qPCR

One µg of total RNA was retro-transcribed using SensiFAST cDNA Synthesis Kit (Meridian Bioscience, Cincinnati, OH, United States). qPCR was carried out in triplicate, using 50 ng of cDNA, SensiFAST SYBR Lo-ROX qPCR mix (Meridian Bioscience) and LightCycler 480 II instrument (Roche, Basel, Switzerland). *GAPDH* was used as a reference gene for the normalization of target gene expression.

The primers used for qPCR are shown below:

for *MEN1*:

Forward Primer: GGAGCTGGCTGTACCTGAAA.

Reverse Primer: GCAATGCCCTTGTGGTAGAG.

for *GAPDH*:

Forward Primer: GAGAAGGCTGGGGCTCATTT.

Reverse Primer: GCAGGAGGCATTGCTGATGA.

*MEN1* and *GAPDH* primers were previously reported in [19] and [20].

### Drug combination analysis

Combenefit software [21] was used for drug combination analysis. The effect of increasing sub-optimal concentrations of MI-136 and EPZ5676 after 9 and 12 days of treatment was calculated by data procession with classical Loewe synergy model.

### RNA sequencing and data analysis

For each sample, 1 µg of total RNA were used for rRNA depletion by using MGIEasy rRNA Depletion Kit (MGI, Shenzhen, Guangdong, China). For validation experiment. Then, the purified RNA was used as an input for indexed libraries preparation using MGIeasy RNA Directional Library Prep Kit V2.0 (MGI) according to manufacturer’s instructions. Equimolar pools were prepared, circularized using MGIEasy Circularization Module (MGI) and subjected to DNA nanoballs generation followed by sequencing (paired-end, 2 × 100 cycles) on the DNBSEQ-G50RS (MGI). For validation experiments, indexed libraries were prepared starting from 1 µg total RNA according to Illumina Stranded Total RNA preparation kit (Illumina Inc., San Diego, CA, USA) and sequenced at a concentration of 1,7 pM on the NextSeq 500 platform (Illumina Inc.). In both cases FastQC tool (http://www.bioinformatics.babraham.ac.uk/projects/fastqc) was used for quality control analysis on generated raw sequencing files (.fastq) followed by adapter trimming using Cutadapt v.4.0 [22]. STAR tool (v.2.7.5a) [23] with the standard parameters was used for alignment of reads on human genome (assembly hg38) considering present in GenCode Release 36 (GRCh38.p12) genes. FeatureCounts [24] and DESeq2 [25] were utilized for expressed genes quantification and differentially expressed genes quantification, respectively. Genes characterized by |Fold-Change|≥Q1 (first quartile) and *padj *≤ 0.05 were considered as differentially expressed.

### Functional analyses and pathway analyses

Investigation of modulated signaling pathways was performed using Ingenuity Pathway Analysis (IPA) and Gene Set Enrichment Analysis (GSEA) [26] tools using the lists of differentially expressed genes. GOplot [27] was used for the Circos plot generation.

### Statistical analyses

Statistical analyses were performed using R (version 4.0.2). Error bars represent means ± SD of independent replicates. Comparisons between two groups were performed by Student’s t-test. Values of *p* ≤ 0.05 were considered as statistically significant.

## Results

### Evaluation of menin expression in OCs

**Fig. 1:**
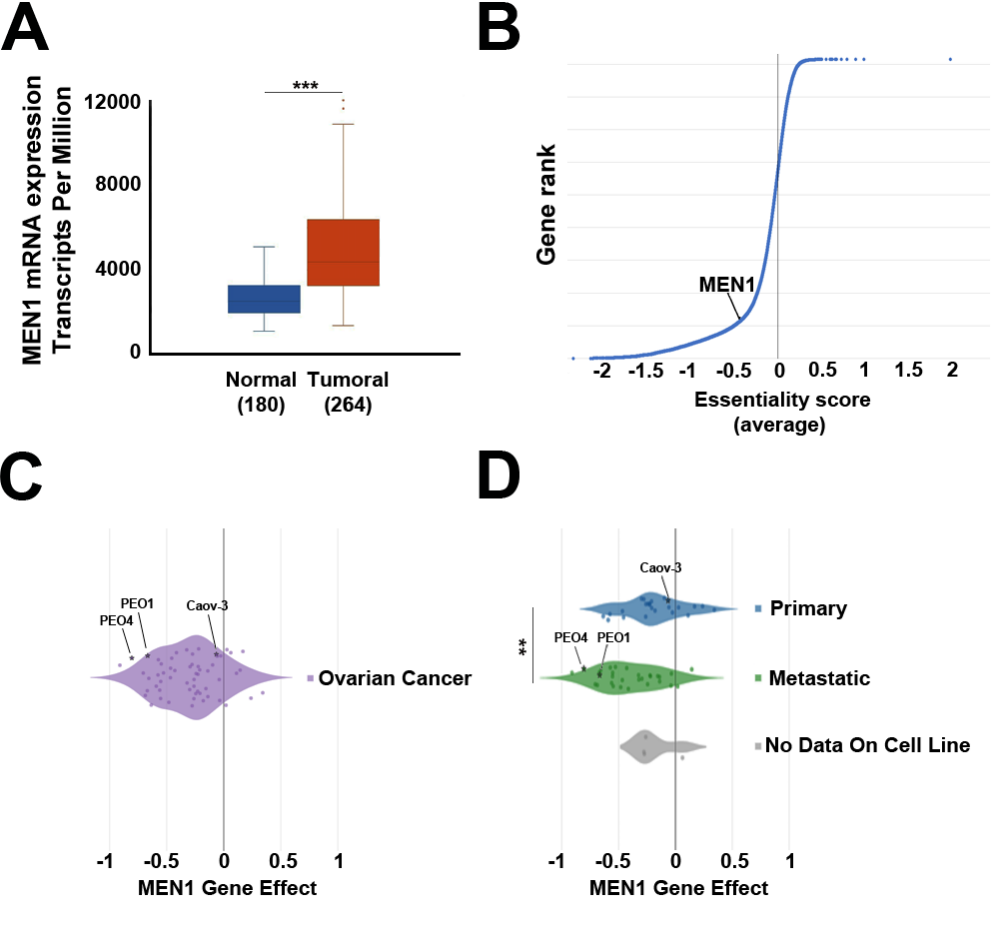
Evaluation of *MEN1* expression and its “fitness” properties in OC cells. (**A**) Box plot showing *MEN1* mRNA expression in ovarian tumors (red) and normal tissues (blue) (*** *p* ≤ 0.0005). (**B**) Rank order plot depicting distribution of standardized essentiality scores (average) for genes screened according to [32] in all OC cell lines. Box plots showing *MEN1* gene essentiality score according to [32] in all OC cell lines (**C**) and in cell lines derived from different tumor types (**D**) (** *p* ≤ 0.005)

Lately, a pro-proliferative role of menin in multiple cancer types including leukaemia, hepatocellular carcinoma, breast, endometrial and prostate cancers has been demonstrated [13], [28], [29], [30], [31]. Moreover, elevated expression of gene encoding for menin protein (*MEN1*) has been found in endometrioid and breast cancers, [30], [11], whereas in hepatocellular and prostate cancer *MEN1* overexpression has been correlated to disease progression [28], [31]. In order to investigate menin role in OC development, we analysed RNA-seq data from ICGC [16] and GTEx expression profiling datasets and found out that *MEN1* expression level is increased in ovarian tumours in comparison to normal tissues (Fig. 1A). We next explored whether menin expression is essential for proliferation and survival of OC cells by interrogating the data of genome-wide CRISPR-Cas9 loss-of-function genetic screen [32], first to place *MEN1* in the context of fitness and non-fitness genes in OC (Fig. 1B), then revealing that menin expression is required for optimal survival for most of analysed OC cell lines (Fig. 1C). Moreover, further analysis demonstrated that cell models isolated from metastatic OC display a statistically significant elevated dependence from menin expression compared to the ones derived from primary tumours (Fig. 1D). Altogether, these results indicate a possible involvement of menin in OC development and progression.

We next adopted, as experimental model, a panel of five cell lines isolated from HGSOC tumours, representing the most frequent, aggressive, and lethal form of OC [33]. Among selected cell lines PEO14 and Caov-3 were isolated from patient before treatment, PEO1 – after the first disease recurrence, whereas OVCAR-3 and PEO4 cells are derived from relapsed tumours established after the acquisition of chemo-resistance. These cell models are characterized by typical HGSOC genetic background since all of them carry pathogenic mutations in TP53, a hallmark genetic aberration found in approximately 96% of such tumours [34], [35]. Moreover, PEO1 cell line bears pathogenic BRCA2 mutation and therefore represents an OC tumour type defective for double stranded DNA repair, known to be associated with hereditary breast and ovarian cancer [36]. Investigation of *MEN1* mRNA and menin protein expression in selected cell lines revealed heterogeneous expression of this molecule with the highest expression level of both of them in PEO1 and PEO4 cells, that we adopted as principal models within the manuscript (Fig. S1A). Interestingly, according to the data of genome-wide CRISPR-Cas9 loss-of-function genetic screen [32], *MEN1* resulted to be a key fitness gene in these cells, indicating that menin represents a plausible therapeutic target in this biological context (Fig. 1C, D).

### Menin silencing induces proliferation reduction and transcriptome deregulation in OC cells

**Fig. 2:**
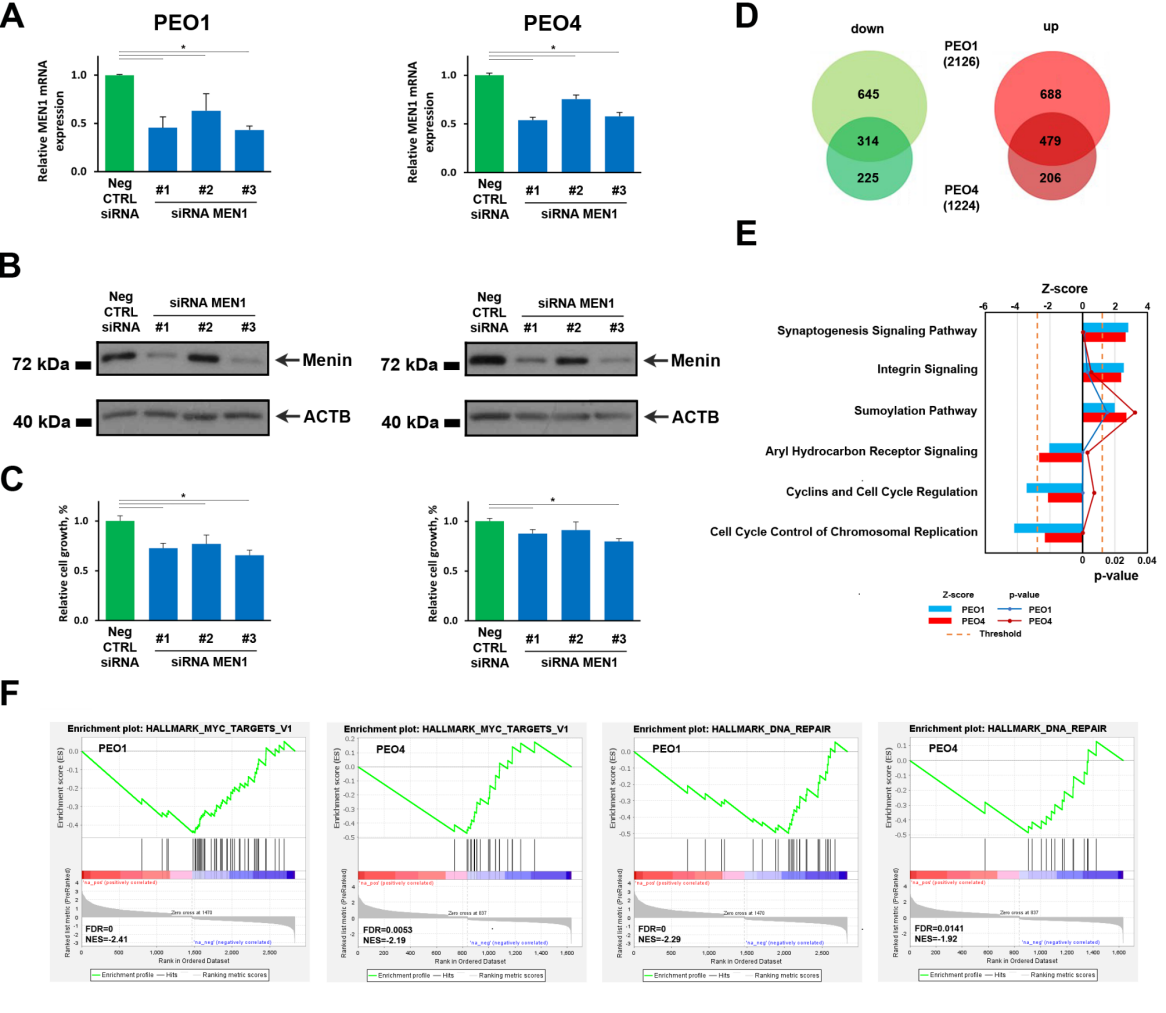
Impact of menin silencing on proliferation and transcriptome of OC cells. Changes of *MEN1* mRNA relative abundance (**A**), menin protein expression (**B**) and cell proliferation (**C**) determined by RT-qPCR, western blot and MTT assay, respectively, in PEO1 (left) and PEO4 (right) cells 96 h post transfection with three siRNAs targeting different regions of the *MEN1* mRNA. Scramble siRNA (CTRL) was used as the negative control. Error bars represent the mean of replicate values ± SD (* *p* ≤ 0.05). (**D**) Venn diagrams showing the number of down- (left) and up- (right) regulated transcripts in PEO1 and PEO4 cells 96 h post transfection with *MEN1*-targeting siRNA #3. RNA-seq was performed in biological duplicates. (**E**) Graphic representation of determined by IPA statistically significant pathways, concordantly deregulated upon *MEN1* silencing in both PEO1 (blue) and PEO4 (red) cells. The dashed orange line marks the Benjamini Hochberg *p*-value (B-H) threshold (0.05). (**F**) Gene set enrichment analysis (GSEA) showing MYC and DNA repair GO terms highlighted by genes, deregulated upon *MEN1* silencing in PEO1 and PEO4 cells. Negative and positive Normalized Enrichment Scores (NES) indicate that down- and up-regulated genes are over-represented. FDR stands for False Discovery Rate

To evaluate menin effect on tumorigenic potential of OC cells, siRNA-mediated *MEN1* knock-down coupled to MTT assay was applied to selected experimental models. Exponentially growing OC cells were transiently transfected with three siRNAs targeting different regions of menin-encoding transcript together with scramble siRNA as negative control. Knock down effect on *MEN1* mRNA, menin protein expression and cell proliferation were evaluated by qPCR, western blot and MTT assay, respectively, 96 hours after transfection (Fig. 2A-C, Additional file 1: Fig. S1B-D). Out of three tested siRNAs, two successfully inhibited mRNA and protein expression in all tested cell lines (Fig. 2A, B, Additional file 1: Fig. S1B, C). Menin depletion inhibited proliferation of all cell lines (Fig. 2C, Additional file 1: Fig. S1D), except for the primary tumour-derived Caov-3 cells, confirming results obtained by CRISPR-Cas9 screening according to which loss of *MEN1* expression does not exert significant impact on Caov-3 cell proliferation (Fig. 1C, D).

Since menin is involved in gene expression control [37], we then focused our attention on analysis of transcriptome changes induced by *MEN1* knock-down in PEO1 and PEO4 cells representing relapsed and chemotherapy-resistant OC tumours and chosen while displaying the highest expression of menin protein. To this aim, from the three *MEN1*-targeting siRNAs, siRNA #3 was chosen, as far as its transfection induced maximum decrease of menin protein expression in all tested cell lines. As shown in Fig. 2D, menin depletion induced profound effect on OC transcriptome since 2126 and 1224 transcripts (Additional file 2: Table S1A and S1B) were found to be deregulated in PEO1 and PEO4 cells respectively (|fold-change| ≥ first quartile (Q1), *padj* ≤ 0.05). Comparison of transcriptome changes between the two cell lines revealed significant similarity since more than a half of genes, differentially expressed in PEO4 cells, were concordantly deregulated in PEO1 (Fig. 2D). Analysis of affected processes by IPA revealed concordant activation of synaptogenesis, integrin and sumoylation pathways and inhibition of aryl hydrocarbon receptor (AhR) and cell cycle-related signalling in both OC cell lines (Fig. 2E). These results were further confirmed and extended by interpretation of gene expression data with GSEA method that highlighted downregulation of MYC and DNA repair-related signalling pathways (Fig. 2F). The results obtained from these two analyses, which are based on different database searches and complement to each other, highlighted common features (Additional file 3: Table S1A and S1B) allowing us to suggest that *MEN1* silencing induces inhibition of cell proliferation by suppression of genes involved in cell cycle regulation. To validate the results obtained here, we employed additional transcriptome sequencing experiments, starting from biological replicate of both cell lines (PEO1 and PEO4) and using a different sequencing technology and platform. As reported in Fig. S2A the correlation between analysed samples resulted very high and differentially expressed genes involved in aryl hydrocarbon receptor (AhR), integrin and cell cycle-related signalling pathways confirmed (Additional file 1: Fig. S2B-D).

### Menin pharmacological inhibition negatively affects OC cell proliferation through deregulation of gene expression

**Fig. 3:**
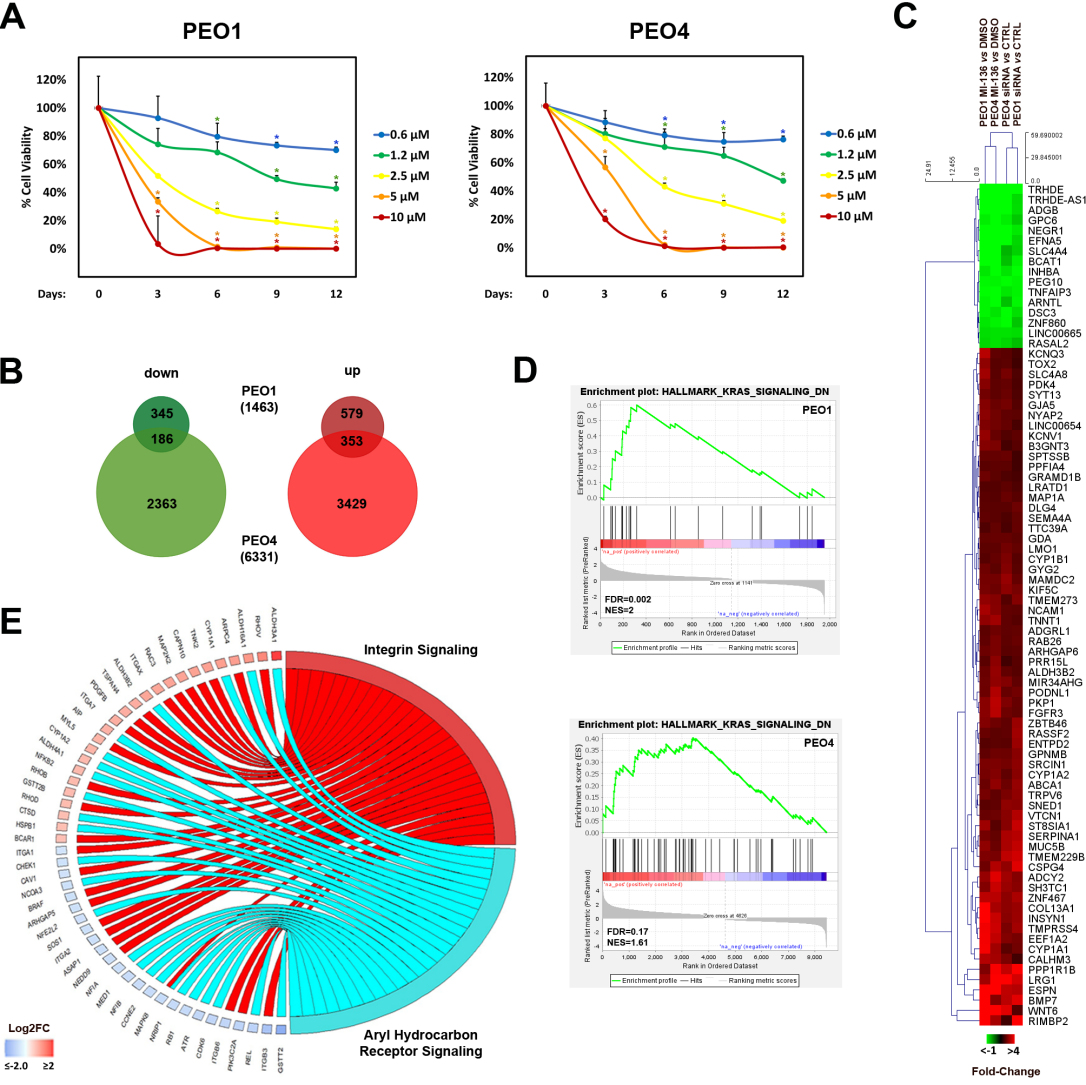
Effects of menin pharmacological inhibition on OC cells proliferation and transcriptome. (**A**) PEO1 (left) and PEO4 (right) relative cell viability assessed in cells treated with increasing concentrations of MI-136. Data represent the mean of six independent replicates ± SD (* *p* ≤ 0.05). (**B**) Venn diagrams showing the number of down- (left) and up- (right) regulated transcripts in PEO1 and PEO4 cells, cultivated in the presence of 0.8 µM MI-136 or vehicle (DMSO) for 9 days. First quartile (Q1) fold-change threshold was applied to identify differentially expressed genes (|FC| ≥ Q1, *padj* ≤ 0.05). RNA-seq was performed in biological triplicates. (**C**) Heatmap and hierarchical clustering with dissimilarity measured using Manhattan distance showing common concordantly deregulated transcripts in PEO1 and PEO4 cells upon *MEN1* silencing or pharmacological inhibition with 0.8 µM MI-136 (|FC| ≥ Q1, *padj* ≤ 0.05). (**D**) Circos plot showing genes, over- (red) and under- (blue) rerepresented in MI-136-treated respect to control (DMSO-treated) PEO4 cells, and the statistically significant (*p* ≤ 0.05) aryl hydrocarbon receptor (AhR) pathway and integrin signaling pathway that tend to be up-regulated

Recent investigations demonstrated that pharmacological blockade of menin activity represents an effective strategy for treatment of acute leukaemia and a subset of solid tumours (reviewed in [38]). Importantly, a number of compounds, inhibiting menin activity *via* disruption of menin interaction with one of its functional partners MLL [39] have been synthesized and some of them are currently being tested in clinical trials [38]. Evaluation of menin blockade effects on the proliferation of the two selected models PEO1 and PEO4 (Fig. 3A) and of the additional OC cell lines (Additional file 1: Fig. S3) revealed that MI-136 treatment induces a profound dose-dependent reduction of cell proliferation. This result was confirmed also for another menin pharmacological inhibitor – MI-503 (Additional file 1: Fig. S4), a compound developed on the basis of MI-136 and characterized by modified molecular scaffold [13].

We next explored transcriptome changes induced by menin pharmacological blockade in PEO1 and PEO4 cells, previously used for analysis of *MEN1* silencing effect on gene expression. As shown in Figs. 3B 9 days-long menin inhibition with MI-136 induced a profound effect on PEO4 transcriptome with 6331 (|fold-change| ≥ Q1, *padj* < 0.05, Additional file 2: Table S1D) deregulated genes and lower impact on PEO1 mRNA profile (1463, |fold-change| ≥ Q1, *padj* < 0.05, Additional file 2: Table S1C). 186 and 353 genes were concordantly down- and up-regulated respectively in both cell lines (Fig. 3B). Moreover, comparison of menin inhibition-induced changes with the ones observed upon *MEN1* knock-down revealed that 16 down- and 66 up-regulated genes, summarized in Fig. 3C, respond in the same way both to menin blockade and silencing in PEO1 and PEO4 cells in statistically significant manner in at least three comparisons indicating that expression of these genes may be regulated by menin-MLL complex. GSEA analysis performed on this subset of differentially expressed transcripts revealed concordant upregulation of KRAS signalling in both cell lines (Fig. 3D), whereas IPA analysis highlighted downregulation of AhR signalling and tendency for upregulation of integrin signalling pathways (Fig. 3E). Interestingly, these pathways were found to be concordantly deregulated also upon *MEN1* silencing (Fig. 2E), suggesting that menin-MLL interaction mediates AhR and integrin signalling pathways activity.

### Combinatorial pharmacological inhibition of menin and DOT1L exerts synergetic effect on proliferation and transcriptome changes in OC cells

**Fig. 4:**
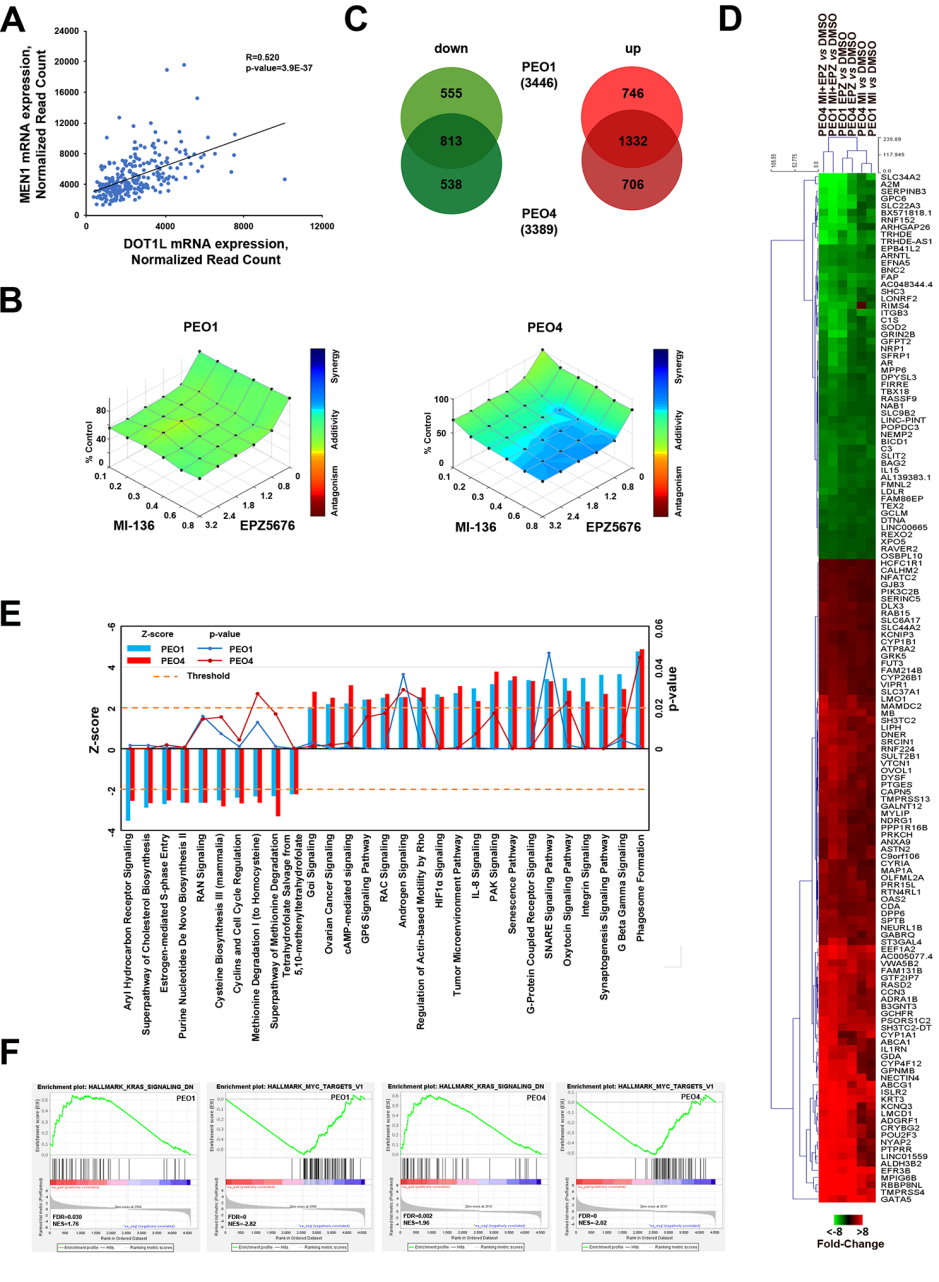
The effects of menin and DOT1L combinatorial targeting on OC cells proliferation and transcriptome. (**A**) Scatter plot showing the correlation of *MEN1* and *DOT1L* mRNA expression in OC tumors. (**B**) D-R Lowe graph showing the effects of combinatorial treatment with increasing MI-136 and EPZ5676 concentrations on PEO1 (left) and PEO4 (right) cells proliferation after twelve days of treatment. (**C**) Venn diagrams showing the number of down- (left) and up- (right) regulated transcripts in PEO1 and PEO4 cells, cultivated in the presence of 0.8 µM MI-136 and 3.2 µM EPZ5676 or vehicle (DMSO) for 9 days (|FC| ≥ Q1, *padj* ≤ 0.05). RNA-seq was performed in biological triplicates. (**D**) Heatmap and hierarchical clustering with dissimilarity measured using Manhattan distance showing common concordantly deregulated transcripts in PEO1 and PEO4 cells upon menin, DOT1L or simultaneous menin and DOT1L pharmacological inhibition with 0.8 µM MI-136, 3.2 µM EPZ5675 or 0.8 µM MI-136 and 3.2 µM EPZ5675 (|FC| ≥ Q1, *padj* ≤ 0.05). (**E**) Graphic representation of statistically significant pathways, identified by IPA analysis, in PEO1 (blue) and PEO4 (red) cells after treatment with the combination of 3.2 µM EPZ5676 and 0.8 µM MI-136 for nine days. (**E**) Graphic representation of determined by IPA statistically significant pathways, concordantly deregulated upon simultaneous menin and DOT1L pharmacological inhibition in both PEO1 (blue) and PEO4 (red) cells. The dashed orange line marks the B-H *p*-value threshold (0.05). (**F**) GSEA showing MYC and DNA repair GO terms highlighted by genes, deregulated upon simultaneous menin and DOT1L pharmacological inhibition in PEO1 and PEO4 cells. Negative and positive NES indicate that down- and up-regulated genes are over-represented

Recently, evidences of complementary activities of menin and DOT1L inhibitors in NPM1-mutant and MLL-rearranged leukaemia have been demonstrated [40], [10]. Moreover, the same result was obtained for estrogen receptor-positive breast cancer cells where synergic effect of pharmacological blockade of these two proteins on the proliferation has been shown and it was highlighted that menin represents DOT1L and ERα co-factor of in this cancer type [11]. Considering the profound effect of DOT1L inhibition on proliferation of PEO1 and PEO4 cells that has been evaluated in our previously published study where we demonstrated that treatment of OC cells with DOT1L inhibitors EPZ004777, EPZ5676 and SGC0946 induced a concentration-dependent inhibition of cell growth in PEO1 and PEO4 cells [4], we investigated whether the interaction between the two proteins occurs also in this cancer type and to estimate the effect of their simultaneous inhibition on proliferation of OC cells. Menin immunoprecipitation performed on nuclear extracts from PEO4 cells, having the higher expression of menin compared to the other selected cell lines, confirmed the presence of DOT1L among co-immunoprecipitated proteins (Additional file 1: Fig. S5). To further confirm the association of these two proteins at chromatin level, we compared the changes induced by MI-136 and EPZ5676 in OC cells (Additional file 2: Tables S1E, S1F) and found out that out of 186 concordantly down- and 353 up-regulated upon MI-136 treatment genes, common for two OC cell lines, 47 and 151 were concordantly down- and up-regulated, respectively also upon EPZ5676 treatment in both cell lines. Similar result was obtained also upon comparison of transcriptional changes induced by EPZ5676 treatment with the effect of *MEN1* silencing that revealed 34 down- and 127 up-regulated transcripts, common for both treatments. Moreover, analysis of RNA-seq data from ICGC [16] expression profiling dataset revealed positive correlation of *MEN1* and *DOT1L* mRNA expression in OC tumours (Fig. 4A) indicating to existence of a subset of OC tumours that expresses both proteins. Next, we tested the effect of a combination of MI-136 with DOT1L inhibitor EPZ5676 that currently undergoes clinical trials (reviewed in [7]). We demonstrated that administration of these two drugs exert an additive effect on the growth of PEO1 cells and on proliferation of chemotherapy-resistant PEO4 cells while using suboptimal concentrations of both drugs (Fig. 4B), with a week synergy observable on the growth of the PEO4 cells upon MI-136 treatment (Fig. 4B, right panel).

To further investigate the molecular mechanisms underlying the interplay of MI-136 and EPZ5676 in OC cells, we analysed transcriptome changes induced by their administration for 9 days, a time point at which the additive effect of the two drugs was already evident (Additional file 1: Fig. S6). We found that also in this case differential expression profiles of the two cell lines (in total 3446 and 3389 deregulated transcripts (Additional file 2: Tables S1G, S1H) with |fold-change| ≥ Q1, *padj* ≤ 0.05 for PEO1 and PEO4, respectively) share significant portion of concordantly deregulated mRNAs that comprised 813 down- and 1332 up-regulated transcripts (Fig. 4C). Further comparison of transcriptional changes induced by combination of MI-136 and EPZ5676 with expression changes induced by these two drugs alone allowed us to determine a subgroup of genes (90 up- and 53 down-regulated) characterized by enhanced deregulation in the presence of drugs combination respect to the single drug-induced expression change (Fig. 4D). Evaluation of the effect on signalling pathway activity induced by the two drugs by IPA and GSEA revealed that AhR, integrin signalling, KRAS, MYC and cell cycle-related pathways, previously found to be affected upon menin silencing or blockade, were affected also in these case (Fig. 4E, F). Moreover, such pathways as IL-8, ovarian cancer and estrogen receptor signalling pathways, previously described to be deregulated upon inhibition of DOT1L alone [4] or in combination with menin pharmacological blockade in other biological contexts [11] were also influenced. Altogether, these results indicate that combinatorial treatment of OC cells with menin and DOT1L inhibitors induces an additive antiproliferative effect on the cell growth, driven by pronounced deregulation of gene expression and signalling pathways.

## Discussion

OC is the most lethal gynaecologic malignancy since more than 75% of affected women are diagnosed at an advanced stage of the disease and less than one-half of patients survive for more than five years after diagnosis. It embraces a heterogenous group of malignancies with different characteristics such as aetiology, molecular biology and others. HGSOC represents the deadliest OC tumour subtype since most patients develop disseminated disease already by the time of diagnosis [41]. Despite standard therapy, that includes cytoreductive surgery followed by platinum-paclitaxel chemotherapy initially gives good response, relapse occurs within two years in around 70–80% of HGSOC patients and eventually almost all recurrent tumours develop chemoresistance, leaving limited therapeutic options for further treatment [42]. Thus, the discovery of novel therapeutic targets including single molecules or signalling pathways, involved in drug resistance and metastasis has become the main focus of the on-going research, representing a promising approach for treatment of these deadly tumours. Among them, epigenetic modulators represent the most promising class of druggable targets due to their potential to effectively reverse transcriptional and epigenetic abnormalities induced by their aberrant activity [43].

In this study we report that targeting *MEN1* gene-encoded protein menin, known as an important DOT1L cofactor in MLL-rearranged leukaemia and antiestrogen therapy-resistant breast cancer cells [10], [7] may represent an effective therapeutic approach against OC. Starting from the observation of increased *MEN1* mRNA expression in OC cells and its relevant essentiality for survival of metastasis-derived cell lines, we demonstrated that siRNA-mediated depletion of *MEN1* expression exerts an antiproliferative effect on the growth of OC cells, that may be explained by inhibition of cell cycle-related signalling and DNA repair obtained by RNA sequencing. Importantly, *MEN1* depletion induced inhibition of activity of MYC proto-oncogene, known to be amplified in around 64% of OC tumours [44] and activated in over half of human cancers including OC [45]. Similar results have previously been observed in fibrosarcoma and liver cancer cells where menin was characterized as a critical cofactor for MYC-mediated transcription, that promotes growth of tumours with deregulated MYC expression [46]. Moreover, in androgen receptor-dependent prostate cancer cells menin is involved in MYC-mediated activation of androgen receptor transcription [47], indicating a cooperative action of the two proteins. Activity of another transcription factor - AhR, was also found to be suppressed upon *MEN1* silencing, whose role in controlling proliferation, migration, and tumour cell invasion has not been extensively determined yet, however there are some indications for its tumour-promoting role in OC since AhR nuclear localization has been associated with worth outcome for OC patients [48] and evidences of AhR receptor role in promotion of cell growth, stemness and metastatic potential of OC [49].

We next demonstrated that pharmacological inhibition of menin-MLL interaction exerts dose-dependent antiproliferative effect on the growth of OC and that also in this case inhibition of AhR signalling may be in charge of the observed effect, indicating the possibility of cooperative regulation of this signalling pathways by menin-MLL complex. Interestingly, we found that MI-136 treatment induced downregulation of KRAS signalling, whose constitutive activation is typically associated with low-grade ovarian carcinoma, endometrioid and mucinous ovarian tumours since these OC subtypes usually bear KRAS activating mutations [50]. However, recent study demonstrated that targeting RAS signalling in HGSOC cells with ADT-006, a small molecular inhibitor of RAS-effector interactions, displayed high sensitivity to this compound *in vitro* [51], indicating an antitumoral effect of KRAS signalling inhibition also in these cells and implying that antiproliferative effect of menin-MLL interaction blockade may be mediated by this signal transduction pathway. Finally, the investigation of the relationship between menin and DOT1L confirmed their functional interplay in OC since nuclear interaction of the two proteins was observed and comparison of transcription profile changes induced by menin or DOT1L pharmacological blockade revealed a subset of commonly deregulated genes. Supporting this result, we demonstrated that simultaneous administration of DOT1L and menin small molecule inhibitors have an additive antiproliferative effect on chemotherapy-sensitive and -refractory relapsed OC cells, and that combinatorial treatment of OC cells with sub-optimal doses of the two drugs induced profound effect on OC transcriptome that included cell cycle, MYC, AhR and KRAS signalling found to be deregulated upon *MEN1* silencing or menin pharmacological blockade.

## Conclusion

The results obtained in this study suggest that menin functionally cooperates with DOT1L in OC cells modulating transcriptome changes involved in key cellular functions including cell proliferation. Combinatorial blockade of DOT1L and menin activity show an additive effect on OC cell growth, thus, we assume that the inhibition of these two epigenetic regulators may represent a worth exploring approach to improve the therapy, response and survival of OC patients. However, it has to be noted that our results provide a starting point in the investigation of DOT1L and menin interplay in OC and further research are needed to determine which of the here discovered functional processes underly the observed antiproliferative effect. Moreover, the mutational status of the cell lines used in this study need to be considered and further validations of DOT1L and menin inhibitors on patient-derived OC models and xenografts are necessary to confirm the beneficial effect of the drug combination in clinically relevant cancer models.

## Electronic supplementary material

Below is the link to the electronic supplementary material.

**Supplementary Material 1: Figure S1.** Impact of menin silencing on proliferation of OC cells. (**A**) RT-qPCR, (left panel) and western blot (right panel) of *MEN1* mRNA and menin expression levels, respectively, in Caov-3, OVCAR-3, PEO1, PEO14 and PEO4 cells. Error bars represent the mean of replicate values ± SD (* *p* ≤ 0.05). Changes of *MEN1* mRNA relative abundance (**B**), menin protein expression (**C**) and cell proliferation (**D**) determined by RT-qPCR, western blot and MTT assay, respectively, in Caov-3 (left), OVCAR-3 (middle) and PEO14 (right) cells 96 hours post transfection with three siRNAs targeting different regions of the *MEN1* mRNA. Scramble siRNA (CTRL) was used as the negative control. Error bars represent the mean of replicate values ± SD (* *p* ≤ 0.05). **Figure S2**. Validation of menin silencing effects on OC cells. **(A)** Correlation coefficient between differentially expressed genes following menin silencing obtained from independent experiments performed with two different sequencing approaches. Graph showing differentially expressed genes belonging to Aryl Hydrocarbon receptor **(B)**, Integrin **(C)** Cyclins and Cell cycle regulation **(D)** signaling pathways obtained from independent experiments performed with MGI and Illumina sequencing approaches. The dashed orange line marks the Fold change threshold (|FC| ≥ Q1, *padj* ≤ 0.05). **Figure S3.** The effects of menin pharmacological inhibition on OC cells proliferation. Caov-3 (left), OVCAR-3 (middle) and PEO14 (right) relative cell viability assessed in cells treated with increasing concentrations of MI-136. Data represent the mean of six independent replicates ± SD (* *p* ≤ 0.05). **Figure S4.** The effects of menin pharmacological inhibition on OC cells proliferation. Caov-3, OVCAR-3, PEO1, PEO14, PEO4 relative cell viability assessed in cells treated with increasing concentrations of MI-136. Data represent the mean of six independent replicates ± SD (* *p* ≤ 0.05). **Figure S5.** Menin associates with DOT1L in nucleus of OC cells. Immunoprecipitation-western blot showing the presence of DOT1L among proteins, co-precipitated altogether with menin from PEO4 nuclear extracts. IgG was used as negative control. **Figure S6.** D-R Lowe graph showing the effects of combinatorial treatment with increasing MI-136 and EPZ5676 concentrations on PEO1 (left) and PEO4 (right) cells proliferation after twelve days of treatment

**Supplementary Material 2: Table S1A**. Differentially expressed transcripts in PEO1 cells transfected with MEN1-targeting siRNA vs transfected with scramble siRNA, determined by stranded RNA-Seq. **Table S1B**. Differentially expressed transcripts in PEO4 cells transfected with MEN1-targeting siRNA vs transfected with scramble siRNA, determined by stranded RNA-Seq. **Table S1C.** Differentially expressed transcripts in PEO1 cells following MI-136 (0.8 µM) treatment for 9 days. **Table S1D.** Differentially expressed transcripts in PEO4 cells following MI-136 (0.8 µM) treatment for 9 days. **Table S1E**. Differentially expressed transcripts in PEO1 cells following EPZ5676 (3.2 µM) treatment for 9 days. **Table S1F.** Differentially expressed transcripts in PEO4 cells following EPZ5676 (3.2 µM) treatment for 9 days. **Table S1G.** Differentially expressed transcripts in PEO1 cells following EPZ5676 (3.2 µM) and MI-136 (0.8 µM) treatment for 9 days. **Table S1H.** Differentially expressed transcripts in PEO4 cells following EPZ5676 (3.2 µM) and MI-136 (0.8 µM) treatment for 9 days

**Supplementary Material 3: Table S2A**. Concordantly deregulated statistically significant signalling pathways determined by IPA following *MEN1* silencing in PEO1 and PEO4 cells. **Table S2B.** Concordantly deregulated statistically significant gene sets determined by GSEA following *MEN1* silencing in PEO1 and PEO4 cells

**Supplementary Material 4**

## Data Availability

Raw RNA sequencing data are deposited in the EBI ArrayExpress database (http://www.ebi.ac.uk/arrayexpress) with accession number E-MTAB-11800. Additional data generated and/or analysed during the current study are available from the corresponding authors upon reasonable request. Uncropped blots from images in the manuscript are included in Supplementary Material 4.

## Abbreviations

AhR: Aryl Hydrocarbon Receptor
BRCA2: Breast cancer type 2 susceptibility protein
CRISPR-Cas9: Clustered Regularly Interspaced Short Palindromic Repeats and CRISPR-associated protein 9
DOT1L: DOT1-like histone methyltransferase
ERα: Estrogen Receptor α
GAPDH: Glyceraldehyde 3-phosphate dehydrogenase
GSEA: Gene Set Enrichment Analysis
GTEx: Genotype-Tissue Expression
HGSOC: High-Grade Serous Ovarian Cancer
ICGC: International Cancer Genome Consortium
IPA: Ingenuity Pathway Analysis
OC: Ovarian Cancer
KRAS: Kirsten Rat Sarcoma virus
MEN1: Multiple Endocrine Neoplasia 1
MLL: Mixed Lineage Leukemia
MTT: 3-(4,5-Dimethylthiazol-2-yl)-2,5-diphenyltetrazolium bromide
MYC: Master Regulator of Cell Cycle Entry and Proliferative Metabolism
NPM1: Nucleophosmin 1
siRNA: Small Interfering RNA
TP53: Tumor Protein p53
