# Supplementary material for: Combinatorial targeting of menin and the histone methyltransferase DOT1L as a novel therapeutic strategy for treatment of chemotherapy-resistant ovarian cancer
Source: Cancer Cell Int. 2022 Nov 4;22:336. doi: 10.1186/s12935-022-02740-6 (PMC9636786; doi:10.1186/s12935-022-02740-6)
Supplement: Supplementary file 1 — Supplementary Material 1: Figure S1. Impact of menin silencing on proliferation of OC cells. (A) RT-qPCR, (left panel) and western blot (right panel) of MEN1 mRNA and menin expression levels, respectively, in Caov-3, OVCAR-3, PEO1, PEO14 and PEO4 cells. Error bars represent the mean of replicate values ± SD (* p ≤ 0.05). Changes of MEN1 mRNA relative abundance (B), menin protein expression (C) and cell proliferation (D) determined by RT-qPCR, western blot and MTT assay, respectively, in Caov-3 (left), OVCAR-3 (middle) and PEO14 (right) cells 96 hours post transfection with three siRNAs targeting different regions of the MEN1 mRNA. Scramble siRNA (CTRL) was used as the negative control. Error bars represent the mean of replicate values ± SD (* p ≤ 0.05). Figure S2. Validation of menin silencing effects on OC cells. (A) Correlation coefficient between differentially expressed genes following menin silencing obtained from independent experiments performed with two different sequencing approaches. Graph showing differentially expressed genes belonging to Aryl Hydrocarbon receptor (B), Integrin (C) Cyclins and Cell cycle regulation (D) signaling pathways obtained from independent experiments performed with MGI and Illumina sequencing approaches. The dashed orange line marks the Fold change threshold (|FC| ≥ Q1, padj ≤ 0.05). Figure S3. The effects of menin pharmacological inhibition on OC cells proliferation. Caov-3 (left), OVCAR-3 (middle) and PEO14 (right) relative cell viability assessed in cells treated with increasing concentrations of MI-136. Data represent the mean of six independent replicates ± SD (* p ≤ 0.05). Figure S4. The effects of menin pharmacological inhibition on OC cells proliferation. Caov-3, OVCAR-3, PEO1, PEO14, PEO4 relative cell viability assessed in cells treated with increasing concentrations of MI-136. Data represent the mean of six independent replicates ± SD (* p ≤ 0.05). Figure S5. Menin associates with DOT1L in nucleus of OC cells. Immunopr [file 12935_2022_2740_MOESM1_ESM.docx]

**Combinatorial Targeting of Menin and the Histone Methyltransferase DOT1L as a Novel Therapeutic Strategy for Treatment of Chemotherapy-resistant Ovarian Cancer Treatment**

Elena Alexandrova, et al.

**Supplementary Figures**

**Figure S1**


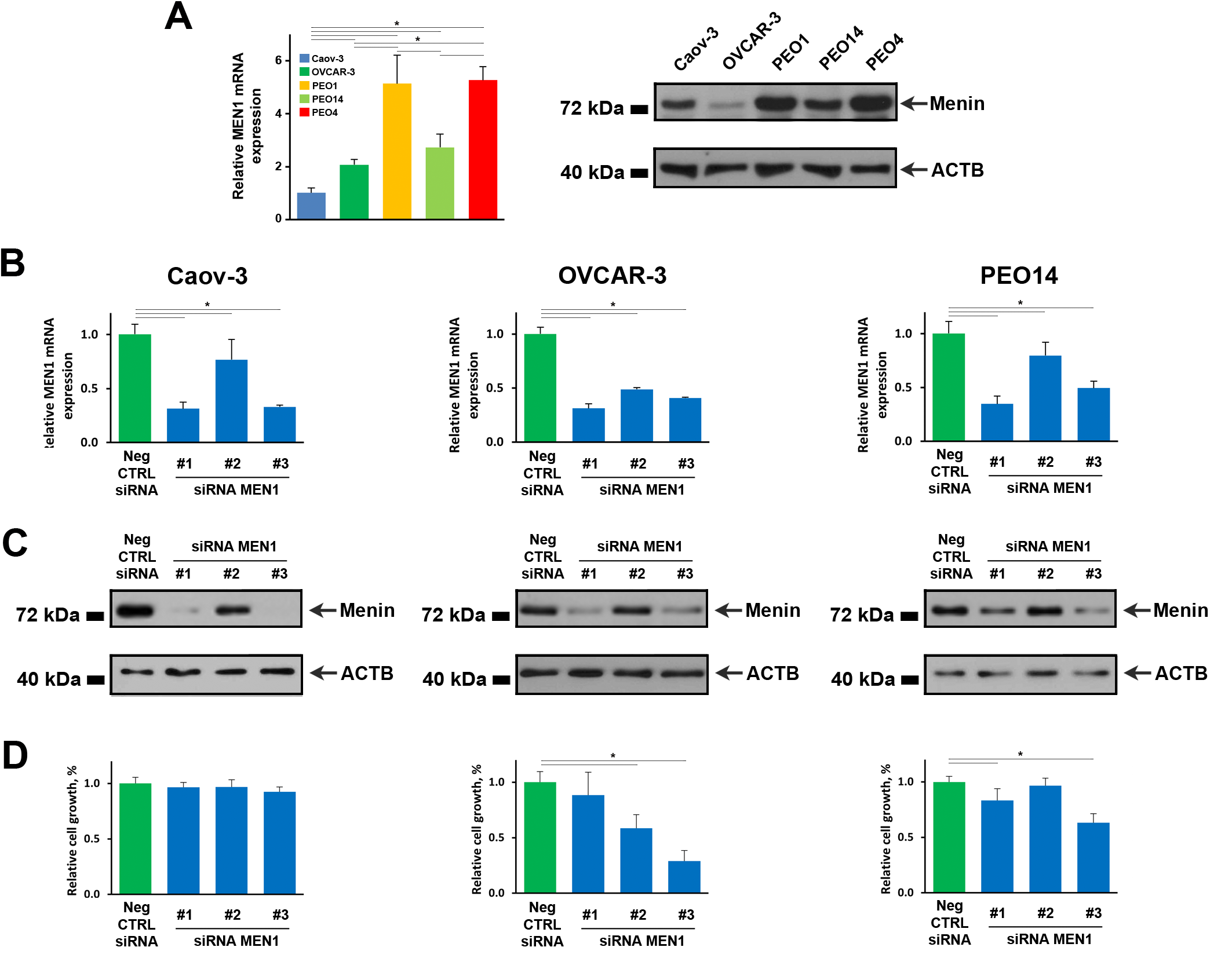


**Figure S1**. Impact of menin silencing on proliferation of OC cells. (**A**) RT-qPCR, (left panel) and western blot (right panel) of *MEN1* mRNA and menin expression levels, respectively, in Caov-3, OVCAR-3, PEO1, PEO14 and PEO4 cells. Error bars represent the mean of replicate values ± SD (* *p* ≤ 0.05). Changes of *MEN1* mRNA relative abundance (**B**), menin protein expression (**C**) and cell proliferation (**D**) determined by RT-qPCR, western blot and MTT assay, respectively, in Caov-3 (left), OVCAR-3 (middle) and PEO14 (right) cells 96 hours post transfection with three siRNAs targeting different regions of the *MEN1* mRNA. Scramble siRNA (CTRL) was used as the negative control. Error bars represent the mean of replicate values ± SD (* *p* ≤ 0.05).

**Figure S2**


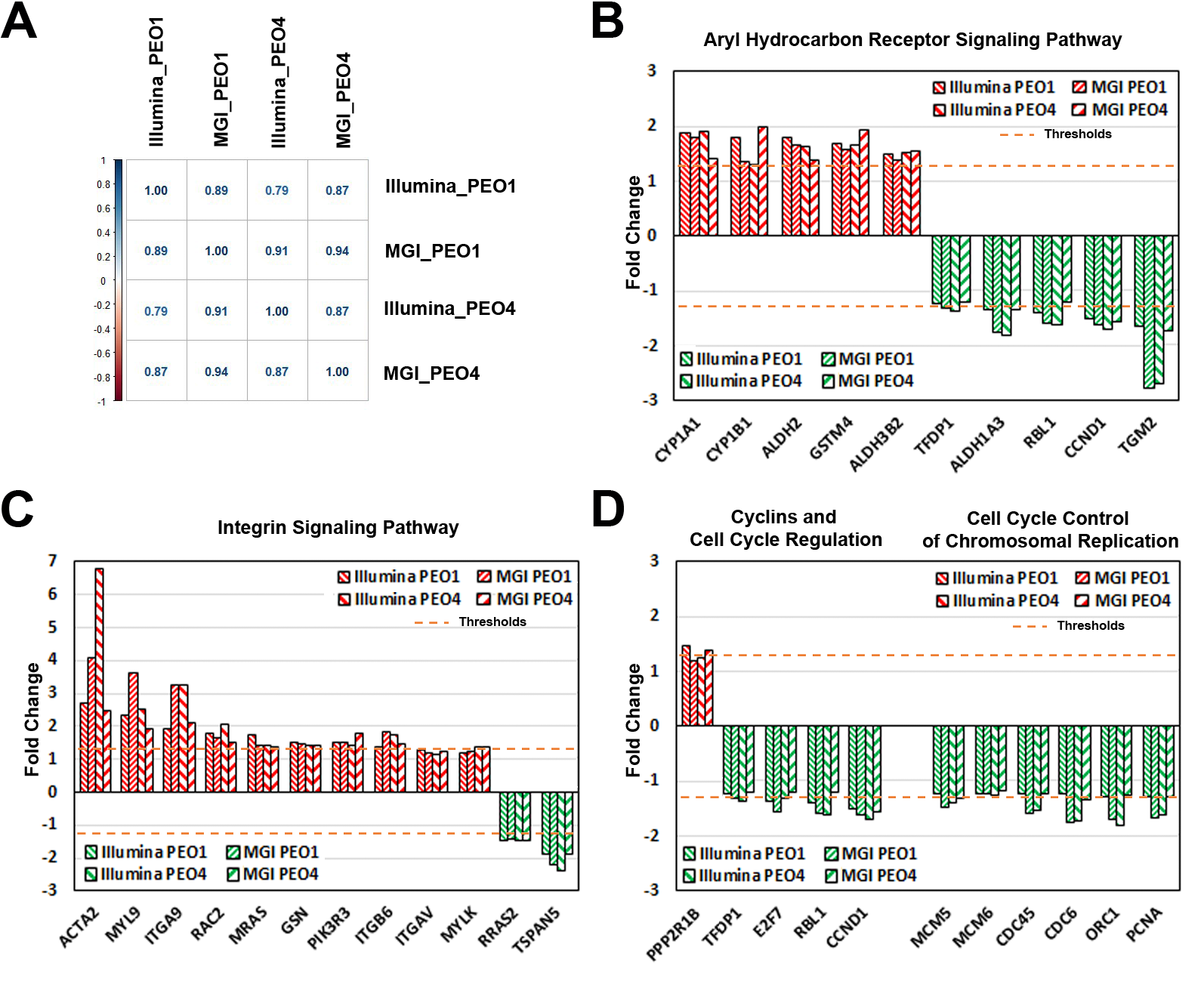


**Figure S2**. Validation of menin silencing effects on OC cells. (A) Correlation coefficient between differentially expressed genes following menin silencing obtained from independent experiments performed with two different sequencing approaches. Graph showing differentially expressed genes belonging to Aryl Hydrocarbon receptor (B), Integrin (C) Cyclins and Cell cycle regulation (D) signaling pathways obtained from independent experiments performed with MGI and Illumina sequencing approaches. The dashed orange line marks the Fold change threshold (|FC| ≥ Q1, padj ≤ 0.05).

**Figure S3**


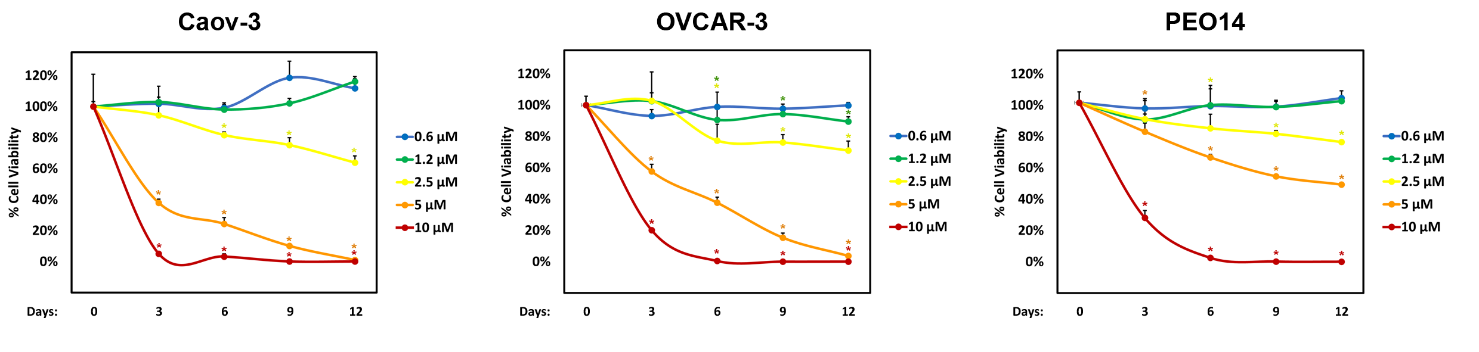


**Figure S3.** The effects of menin pharmacological inhibition on OC cells proliferation. Caov-3 (left), OVCAR-3 (middle) and PEO14 (right) relative cell viability assessed in cells treated with increasing concentrations of MI-136. Data represent the mean of six independent replicates ± SD (* *p* ≤ 0.05).

**Figure S4**

**
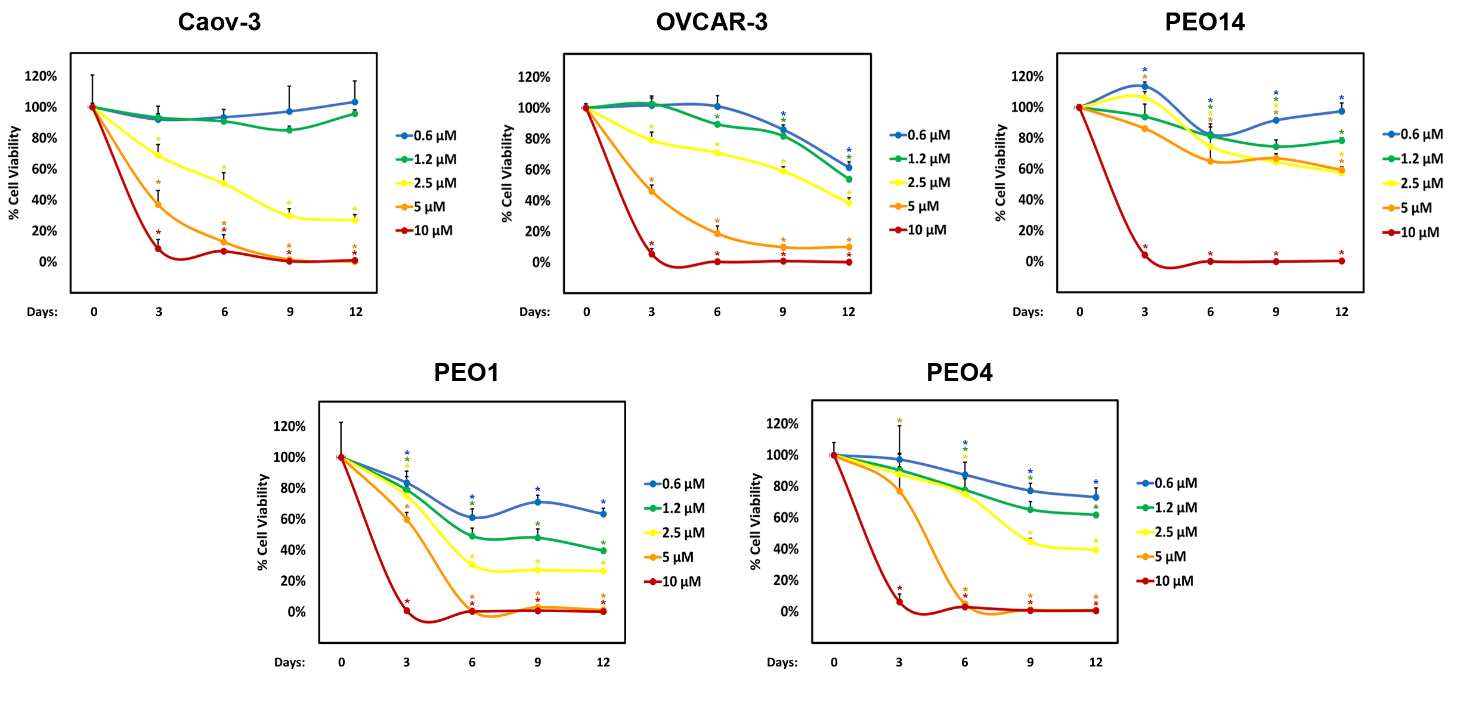
**

**Figure S4.** The effects of menin pharmacological inhibition on OC cells proliferation. Caov-3, OVCAR-3, PEO1, PEO14, PEO4 relative cell viability assessed in cells treated with increasing concentrations of MI-136. Data represent the mean of six independent replicates ± SD (* *p* ≤ 0.05).

**Figure S5**

**
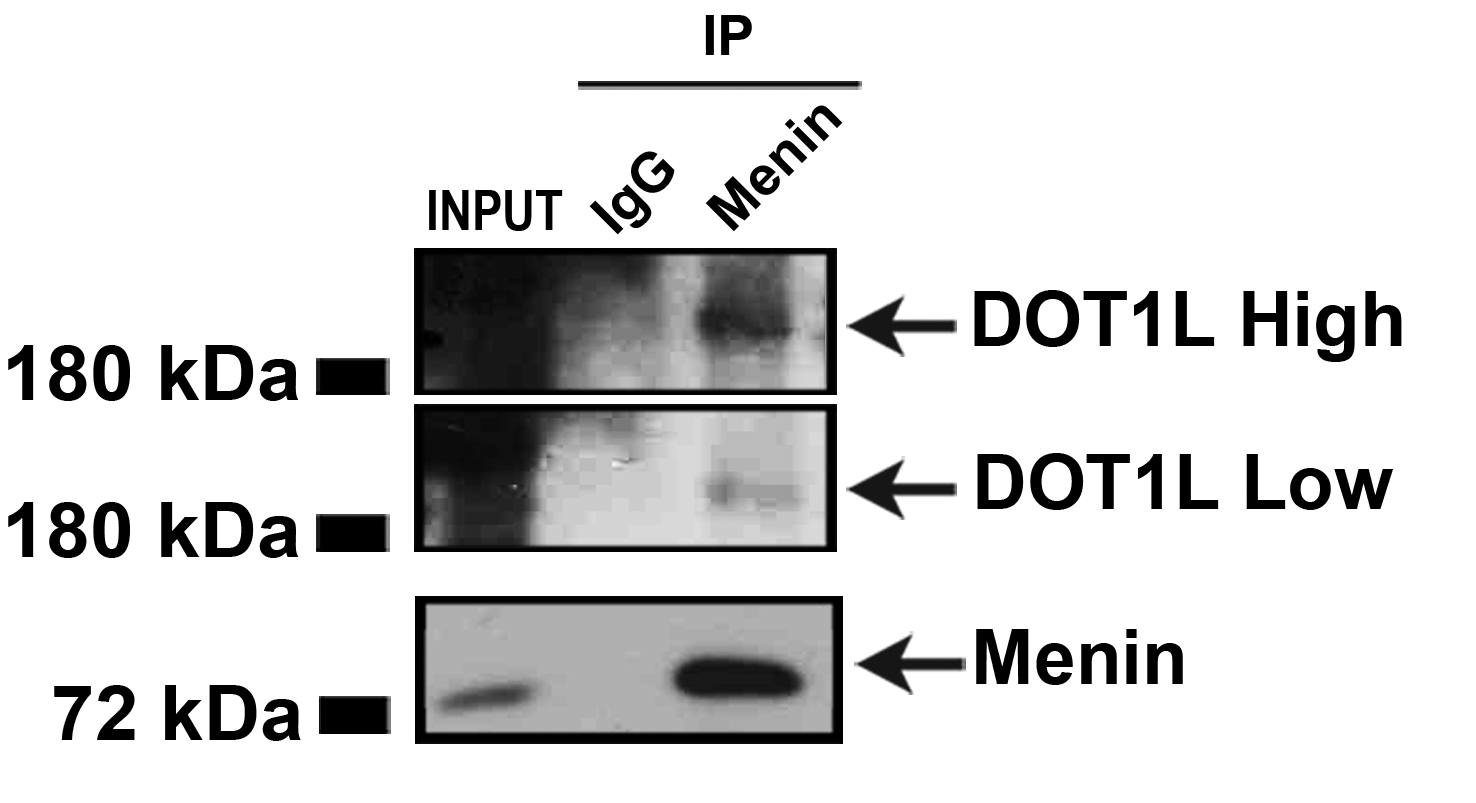
**

**Figure S5.** Menin associates with DOT1L in nucleus of OC cells. Immunoprecipitation-western blot showing the presence of DOT1L among proteins, co-precipitated altogether with menin from PEO4 nuclear extracts. IgG was used as negative control.

**Figure S6**


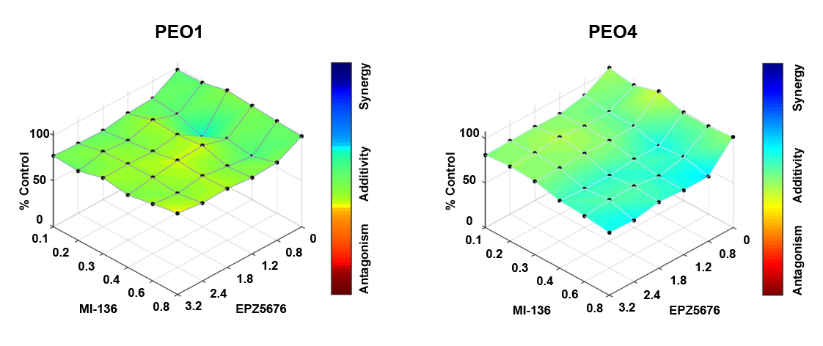


**Figure S6.** D-R Lowe graph showing the effects of combinatorial treatment with increasing MI-136 and EPZ5676 concentrations on PEO1 (left) and PEO4 (right) cells proliferation after twelve days of treatment.
