# Supplementary material for: Combinatorial targeting of menin and the histone methyltransferase DOT1L as a novel therapeutic strategy for treatment of chemotherapy-resistant ovarian cancer
Source: Cancer Cell Int. 2022 Nov 4;22:336. doi: 10.1186/s12935-022-02740-6 (PMC9636786; doi:10.1186/s12935-022-02740-6)
Supplement: Supplementary file 4 — Supplementary Material 4 [file 12935_2022_2740_MOESM4_ESM.pdf]

Uncropped blots Fig. 2B (MEN1)

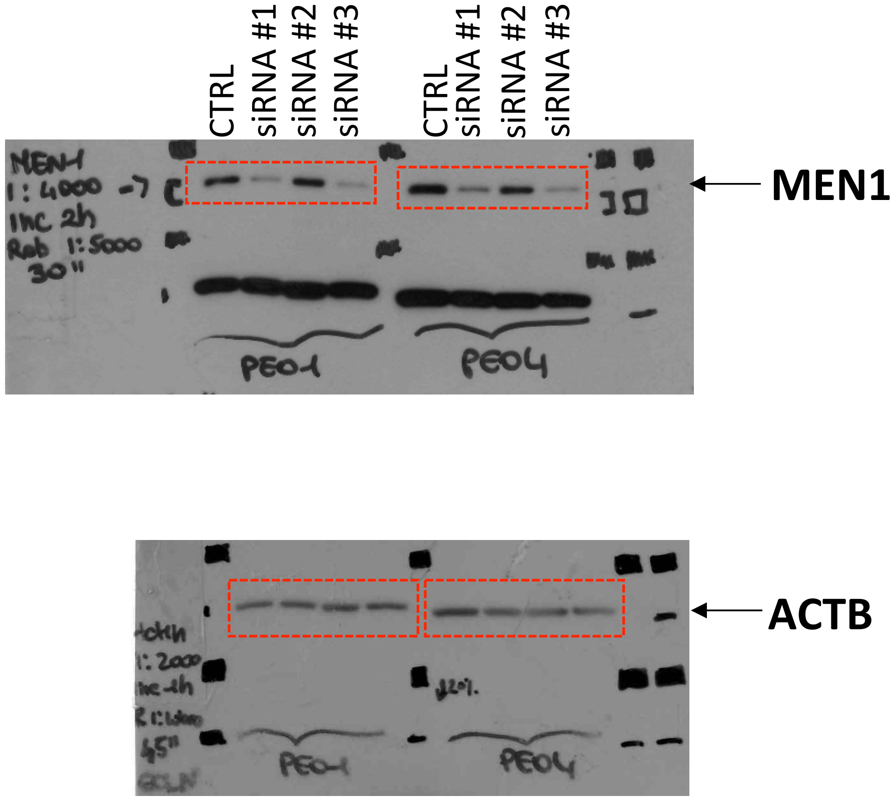

Uncropped blots Fig. S1A (MEN1)

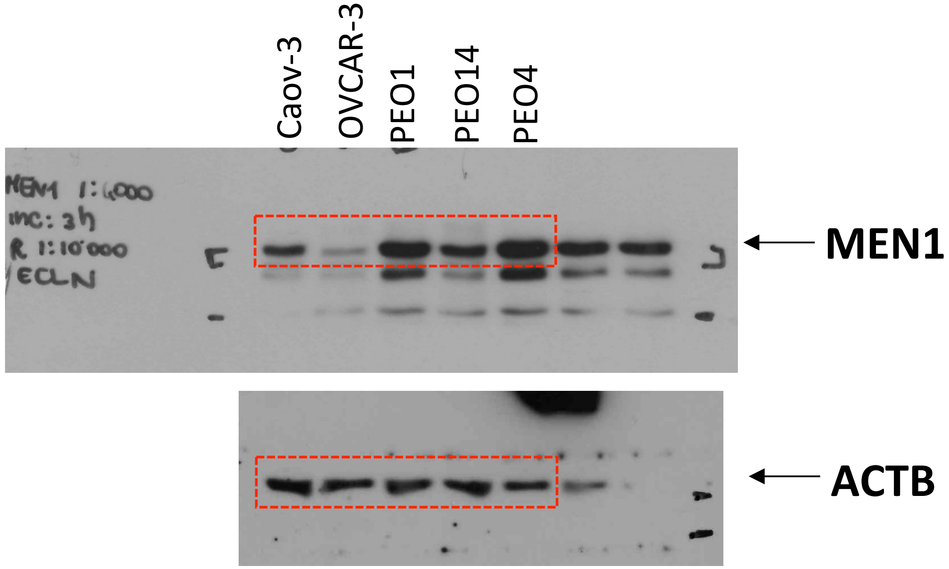

Uncropped blots Fig. S1C (MEN1)

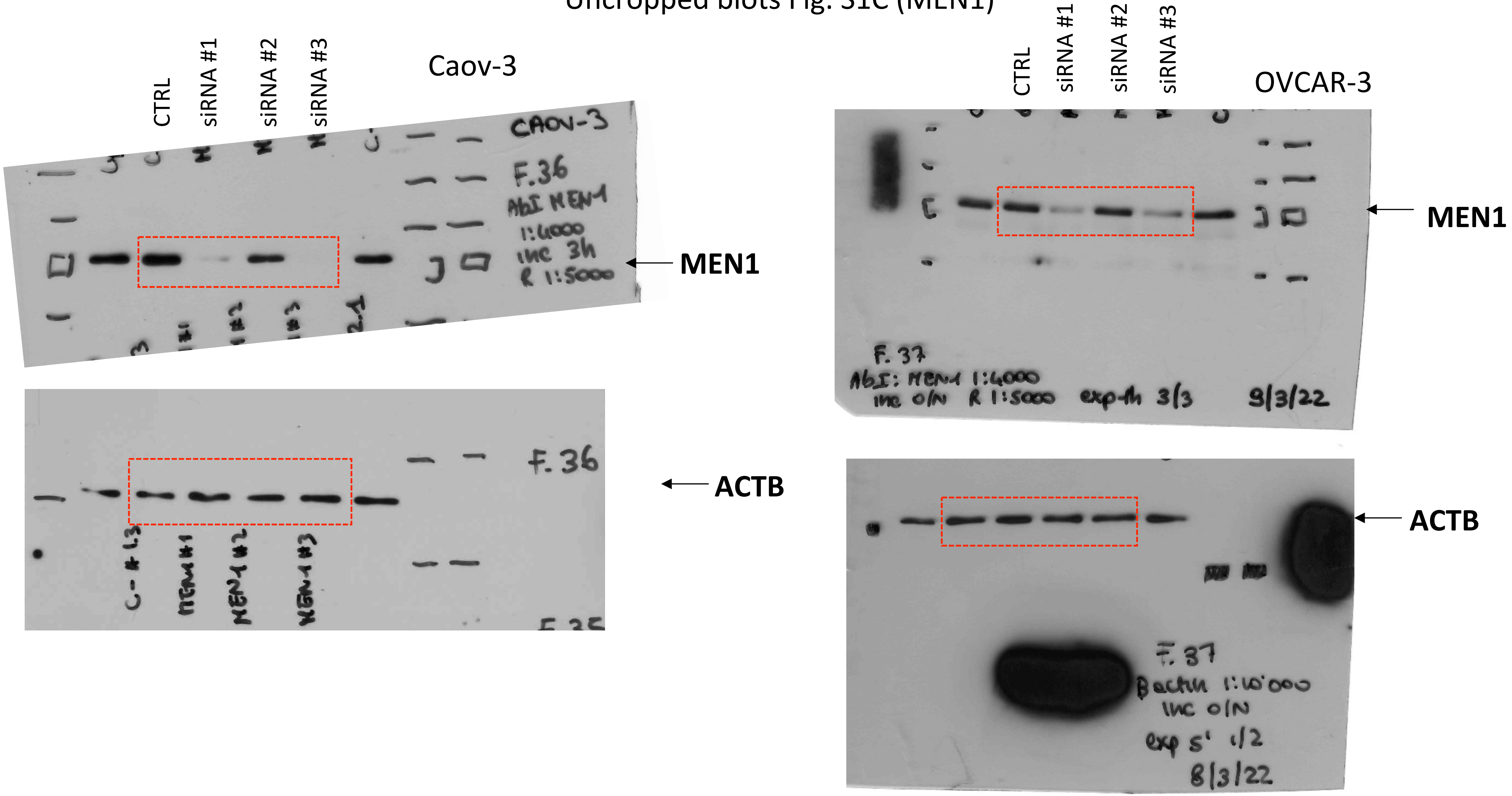

Uncropped blots Fig. S1C (MEN1)

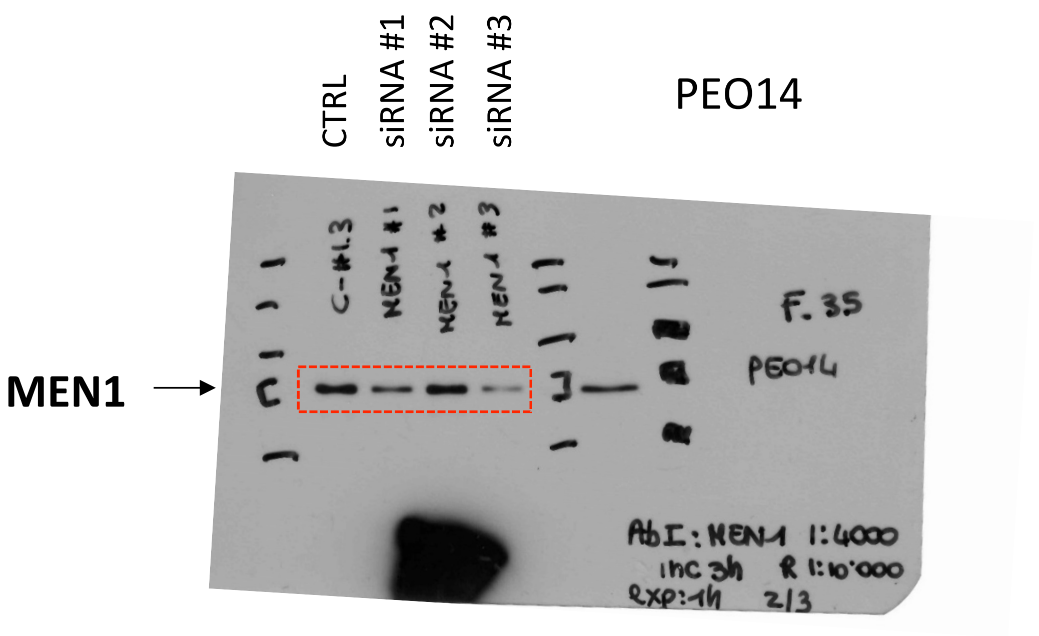

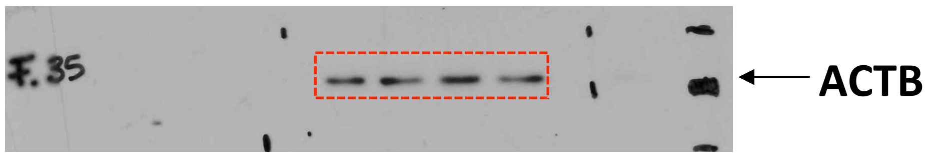

Uncropped blots Fig. S5

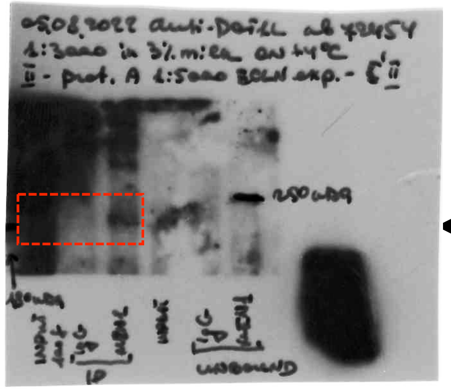

DOT1L high exposition

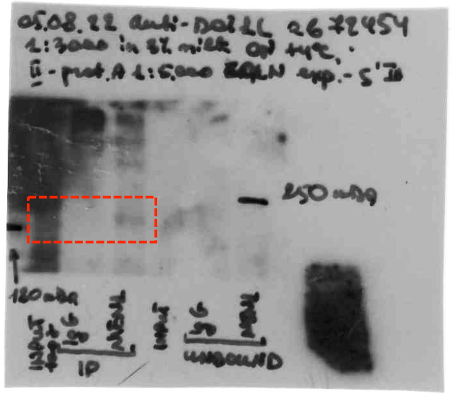

DOT1L low exposition

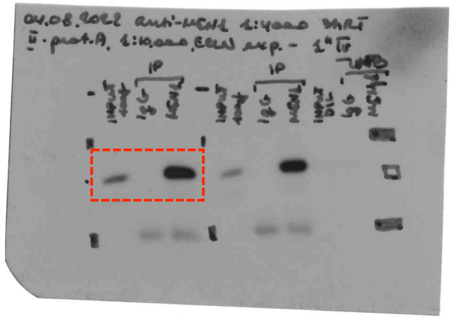

MEN1
